# Know and use your personal strengths! A Spanish validation of the strengths knowledge and use scales and their relationship with meaningful work and work-related well-being

**DOI:** 10.3389/fpsyg.2023.1086510

**Published:** 2023-04-20

**Authors:** Josefina Peláez Zuberbühler, Cristián Coo Calcagni, Marisa Salanova

**Affiliations:** WANT Research Team, Universitat Jaume I, Castelló de La Plana, Spain

**Keywords:** personal strengths, meaningful work, work engagement, mental health, well-being, scale validation

## Abstract

**Introduction:**

Research in the field of work and organizational psychology increasingly highlights the role of meaningful work as a protector of well-being at work. This study tests the role of strengths knowledge and use as new pathways through which meaningful work may have a positive effect on work engagement and mental health.

**Methods:**

Study 1 and Study 2 report the validation of the Spanish Strengths Use and Knowledge Scales respectively, with samples of *N* = 617 (Study 1) and *N* = 365 (Study 2) employees. Study 3 tests the mediating effects of strengths use and knowledge in a model with different work-related constructs in another sample of *N* = 798 employees.

**Results:**

Findings from Studies 1 and 2 indicate that the instruments offer adequate evidence of reliability and validity. Results from Study 3 revealed that strengths knowledge is a mediator in the relationship between meaningful work and strengths use. Findings also confirmed the mediating roles of strengths use in the relationship between meaningful work and work engagement, and between meaningful work and mental health.

**Discussion:**

This study highlights the ability to be aware of and apply signature strengths as effective and novel pathways to foster well-being at work through the cultivation of meaningful work.

## 1. Introduction

Positive psychology is defined as the scientific study of the optimal functioning of individuals and organizations (Seligman and Csikszentmihalyi, 2000). Its aim is to increase positive emotions, attitudes, and behaviors, rather than minimizing negative outcomes such as weaknesses and pathologies. This renewed emphasis, focusing on the positive aspects of human experience, has led to an increase in the research into the concept of strengths, whether defined as character strengths (Niemiec, 2020) or more general applications of this construct in different settings (i.e., work and life; Heintz and Ruch, 2020; Huber et al., 2020).

Indeed, the strengths approach is one of the main pillars of positive psychology, playing a crucial role in human functioning and flourishing (Peterson and Park, 2009). According to this approach, everyone naturally possesses a series of signature strengths, and the personal investment in the identification, use and development of these strengths can foster positive outcomes such as well-being, work engagement and optimal functioning (Dubreuil et al., 2016; Huber et al., 2020). This investment is particularly important in times of convulsions and changes, where uncertainty is an important life stressor, as has been the case for the COVID-19 pandemic during the last couple of years. The worldwide spread of this pandemic outbreak triggered drastic changes with consequent health and economic crises. As a result, the social and work environments are changing rapidly, affecting workers’ physical and mental health (Brooks et al., 2020). Thus, now more than ever it is necessary to explore new paths toward the cultivation of mental health and well-being at work.

Previous research shows that workers who experience their jobs as meaningful report higher levels of well-being (Steger, 2019). Considering this issue, and based on the Job Demand-Resources model (Bakker and Demerouti, 2017), it is crucial to explore new pathways to cultivate meaning and well-being at work. Based on a stepwise approach toward achieving this goal, the aim of this study is twofold. First, we intend to validate and adapt strengths knowledge and use scales (Govindji and Linley, 2007) to the Spanish and Latin American context. Second, we seek to explore the mediating roles of strengths knowledge and strengths use in the relationship between meaningful work and well-being (i.e., work engagement and mental health).

## 2. Theoretical background

### 2.1. Strengths knowledge and use

Based on the previous literature, a strength can be defined as a natural capacity for behaving, thinking, and feeling that is authentic and energizing for the individual, and enables optimal functioning and development (Govindji and Linley, 2007). In the early years of positive psychology, Seligman (2004) defined character strengths as morally valued traits whose use contributes to fulfilment and happiness. The author and his colleagues proposed a taxonomy of strengths, known as the Values in Action-Inventory of Strengths (*VIA*-IS; see Peterson and Seligman, 2004, for a review), with the aim of providing the field with a common vocabulary and a direction for research and application for the enhancement of human potential. The *VIA*-IS consist of 24 different strengths, such as creativity, leadership, or humor, classified under six broad virtues (i.e., wisdom, courage, love, justice, temperance, and spirituality). Research has shown that these 24-character strengths are indeed universal, given the fact they are remarkably consistent in their endorsement across cultures (McGrath, 2015; Niemiec, 2019). Researchers have previously linked strengths to a sense of self, identity, and authenticity that usually leads to a strong intrinsic motivation to put them into practice (Harzer, 2020).

Previous studies used ‘strengths knowledge’ and ‘strengths use’ as possible active ingredients of the character strength approach (Duan et al., 2019). Strengths knowledge refers to the awareness and recognition of one’s strengths, whereas the use of a given character strength refers to the extent to which specific circumstances allow an individual to apply strengths in a variety of settings (Harzer and Ruch, 2013). In the words of Linley and Harrington (2006), p. 41 ‘strengths are natural, they come from within, and we are urged to use them, develop them, and play to them by an inner energizing desire’. These authors stated that the use of signature strengths leads individuals to feel good about themselves, enhancing their ability to do what they naturally do best, and work toward fulfilling their potential.

Character strengths knowledge and use are beneficial, because they are connected to multiple positive effects on health and well-being. There is a growing body of research, related to the relationship between character strengths and various situational, personal, and contextual variables (Proctor et al., 2011). Although research on strengths knowledge and use is still in its childhood, the number of studies examining its association with related outcomes in different settings (i.e., life, education, and work) is increasing (Niemiec, 2018; Duan et al., 2019). From a general ‘life’ perspective, the identification and use of character strengths can lead, among others, to higher levels of psychological well-being (Niemiec, 2019), life satisfaction (Douglass and Duffy, 2015), and mental health (Duan, 2016). The strengths-based approach is also emerging in the organizational context. In a previous study with a heterogeneous sample of employees, Bakker and van Woerkom (2018) found that strengths use on a weekly basis led to work engagement. Moreover, emphasizing personal strengths in the workplace helps employees to achieve their goals more effectively and perform better (Dubreuil et al., 2014).

Given the growing amount of research on strengths in different settings, their identification and application are becoming potentially important tools in personal and organizational development, and these tools are increasingly attractive for practitioners (Ruch et al., 2020). More and more professionals (i.e., therapists, coaches, and consultants) are using strengths-based interventions (i.e., identification, use and development of character strengths) with their customers (Schutte and Malouff, 2019) because they have found this type of intervention to be significantly associated with increments in well-being (Dubreuil et al., 2016), work engagement and job performance (Peláez et al., 2020; Peláez Zuberbühler et al., 2020), as well as with a decline in negative emotional symptoms (Duan et al., 2019).

Previous studies on strengths measurement validation have focused on assessing the degree in which people possess different strengths, such as the *VIA*-IS (Ruch and Proyer, 2015). There is a growing body of research, dedicated to the examination of such classification systems for character strengths. However, there remains a limited amount of research, examining generic strengths use (Bakker and van Woerkom, 2018). While possessing higher levels of a particular strength, in comparison with other individuals, may lead to well-being, recent evidence indicates that is the largest benefits come from creating knowledge and applying strengths (Huber et al., 2020). In the last decade, different instruments have been developed to measure the application of strengths. One example is the ‘Applicability of Character Strengths Rating Scales’, which aims to measure the extent to which each of the 24-character strengths of the *VIA*-IS is applicable in work and private life (Harzer and Ruch, 2013; Huber et al., 2020).

Taking into consideration the void in the study of strengths knowledge and use, Govindji and Linley (2007) developed the ‘Strengths Knowledge Scale’ (SKS) and the ‘Strengths Use Scale’ (SUS) for a more generic assessment of the knowledge and use of all individual kinds of strengths in a general adult population, providing a preliminary empirical basis for their validation. The original English version of the SUS (Govindji and Linley, 2007) has already been translated into different languages, such as Hebrew (Littman-Ovadia et al., 2017) and German (Huber et al., 2017), in both cases exhibiting good validity and reliability. Additionally, work-adapted SUS versions have been developed in English (Kong and Ho, 2016) and French (Dubreuil et al., 2014), and a strengths-based climate scale, partially built upon the SKS and the SUS (Govindji and Linley, 2007), was developed and validated in the Dutch and Belgian contexts (van Woerkom and Meyers, 2015).

Despite the increasing research interest in the development and examination of strengths knowledge and use, to date, no rigorous psychometric evaluation has been carried out in Spanish-speaking countries. Moreover, the availability of these scales in contexts of crisis and uncertainty, such as those generated by the COVID-19 pandemic, is more important than ever if we seek to effectively mitigate some of the negative effects the pandemic has brought upon society. Therefore, the first aim of the present study is to fill this void by examining the psychometric properties, including factorial validity, reliability, and cultural invariance, of the SUS and the SKS in two different studies (Study 1 and Study 2 respectively) with samples of Spanish and Latin American workers. To examine reliability, both studies further explore the relationships between strengths knowledge and strengths use, and several measures of well-being, (e.g., psychological capital, mental health, happiness). With these two studies we aim to contribute to literature by exploring the application of individual strengths in new cultural Spanish-speaking contexts during the COVID-19 pandemic. Moreover, while recent studies in other cultural contexts only validated the SUS, we go one step further and consider both SKS and SUS. Previously, researchers sustained that a necessary precondition for applying character strengths is to gain awareness of one’s strengths and to know when one is at his/her best (Duan et al., 2019). Therefore, to address and apply strengths in different settings, it is important to assess both strengths knowledge and strengths use, as well as the relationship between both. From a practical perspective, we aim to contribute to the strengths-based approach with two validated measures to effectively assess these constructs and develop effective and quality strengths-based interventions within the organizational context in the Spanish-speaking population.

### 2.2. Meaningful work, mental health, and work engagement

The WHO (2018) has determined that mental health is more than just the mere absence of mental disorders or disabilities, and refers to a ‘a state of well-being in which the individual realizes his or her own abilities, can cope with normal stresses of life, can work productively and fruitfully, and is able to contribute to his or her community’. In the organizational field, a negative and stressful working environment may lead to mental health disorders, such as psychological distress, anxiety, and depression, which are the main contributing factors to loss of productivity and absence due to illness (Leka and Nicholson, 2019). On the other hand, workplaces that promote positive mental health and well-being are more likely to reduce absenteeism, increase productivity, and benefit from associated financial gains (Ruhle et al., 2020). Another paramount positive variable related to well-being and of great significance for both employees and organizations, is work engagement. This positive state of mind is characterized by three dimensions: (1) vigor: which refers to high levels of energy and mental resilience, the willingness to invest effort in one’s work, and persistence in facing difficulties; (2) dedication: which refers to strong involvement, that is, psychological identification with one’s work, and characterized by a sense of significance, enthusiasm, pride, inspiration, and challenges; and (3) absorption: which refers to a state of complete concentration and being engrossed in one’s activities (Schaufeli et al., 2002).

The crisis, caused by the Covid-19 coronavirus pandemic, is affecting all working environments worldwide. The current health, social, and economic changes have required employees to exert more effort, apply different skills, and work under greater physical and mental pressure. As a result, higher levels of exhaustion, anxiety, absenteeism, and lower levels of productivity have been reported in most work settings (Brooks et al., 2020). Thus, now more than ever, it is necessary to explore new paths to cultivate mental health and well-being at work. Previous research has emphasized the important role of meaningful work as an important protector of well-being at work, especially in times of crisis such as the Covid-19 pandemic, in which the face of business and work life has been altered completely (Correia and Almeida, 2020).

Steger (2019), p. 218 defined meaningful work as ‘work that people gladly, gratefully, and energetically give their best self and effort to’. The author and his colleagues refer to meaningful work not simply as the meaning that people attribute to work, but as work that is significant and positive in valence (meaningfulness), that fosters personal growth, and contributes to one’s overall life purpose and to a greater good beyond oneself (Steger et al., 2012). Employees who consider their work meaningful express a higher level of meaning in everything, from career to personal life, and a greater contribution to the world around them (Steger, 2019). They tend to experience work as meaningful when they feel that their work is notable and supportive of the organization’s goals and achievements. This makes meaningful work a vital job resource and characteristic that can predict important employee outcomes (Ahmed and Ismail, 2020). Employees who sense their work is meaningful report greater well-being (Lepisto and Pratt, 2017), view their work as more central and important (Steger et al., 2012), and report higher levels of job satisfaction (Allan et al., 2019). Previous research has also demonstrated the predictive value of meaningful work in employee engagement. In this respect, the set of attitudes that constitute meaningful work should facilitate employees’ abilities to invest themselves and engage more fully in their work (Steger et al., 2013). A recent meta-analysis underlined a strong association of meaningful work with the cultivation of work engagement, among other positive work outcomes such as commitment, a sense of meaning in life, and general health (Allan et al., 2019).

### 2.3. The mediating role of strengths knowledge and strengths use

Meaningful work may increase mental health and well-being through various paths, such as buffering the impact of work stress and improving people’s purpose in life. However, several lines of inquiry suggest that meaningful work alone is not enough (Allan et al., 2018). People who experience their work as meaningful but are unable to fully employ their skills and abilities may be particularly at risk for lower levels of well-being (Allan et al., 2020). On the contrary, people who sense their work is meaningful and possess qualities that are desirable to organizations report greater well-being and job satisfaction (Steger et al., 2012). Research has demonstrated that one of the consequences of individuals sensing their work is meaningful, is their ability to use their strengths at work (Arnold et al., 2007; Steger et al., 2013). This suggests an interactive effect, in which work must not only be meaningful, but also provide the opportunity to discover and use personal resources, such as character strengths, to achieve benefits for mental health and enhance work engagement. However, to date, research exploring character strengths as underlying mechanisms is still scarce.

Recent research has demonstrated how applying and developing strengths is an effective positive-psychology approach to increase a feeling of thriving and decrease negative emotional symptoms, thus leading to overall better mental health (Duan et al., 2019). Previous studies demonstrated that having greater knowledge about strengths, as well as more confidence in using them, leads to higher levels of subjective and psychological well-being (Niemiec, 2019). The application of the strengths use construct in the specific organizational context has demonstrated that its use and development can foster positive experiences at work, such as work engagement (Harzer and Ruch, 2013; Bakker and Van Wingerden, 2021). Thus, creating awareness for individual character strengths, as well as enabling its applicability, is a promising approach to increase well-being and job performance (Huber et al., 2020).

Framed within the JD-R model (Demerouti et al., 2001; Bakker and Demerouti, 2007), the role of character strengths has been highlighted as a personal resource (Harzer, 2020). The JD-R model states that job characteristics can be classified in two main categories, namely job demands–aspects of work that cost energy and create strain–and job resources–aspects of work that help employees to deal with demands and achieve their goals. This approach has recently integrated personal resources into the model that refer to the beliefs people hold regarding the control they have over their (work) environment. While job demands may initiate a health-impairment process if there is exposure to daily workload over a long period of time, job and personal resources initiate a motivational process, contributing positively to work engagement (Bakker and Demerouti, 2018). The application of character strengths has been considered as the fit between personal resources and job resources, given that the job allows and fosters the use of signature strengths. According to the JD-R model, employing one’s signature strengths may foster feelings of excitement, as its application is energizing and invigorating, leading to a motivation process from which work engagement arises, which in turn leads to desirable work outcomes such as life satisfaction, organizational commitment, and higher job performance (Harzer, 2020).

A variety of studies have analyzed the positive link between meaningful work and well-being variables such as work engagement and mental health (Allan et al., 2019; Steger, 2019). Similarly, research on the link between strengths knowledge, strengths use, and well-being is on the rise (Huber et al., 2020). However, research on the ability to discover and use personal strengths as underlying mechanisms that explain how meaningful work exerts its influence on well-being-related outcomes in the workplace is still scarce. Results from a recent study demonstrated that perceiving their work as meaningful motivates employees to use their strengths, using their strengths makes them feel authentic and efficacious, and these positive psychological states fuel work engagement (Van Wingerden and Van der Stoep, 2018). Despite these interesting findings, there is still need to investigate the roles of both strengths knowledge and strengths use as mediators in the link between meaningful work, work engagement and mental health.

We hypothesize that strengths use will act as a mediator between meaningful work and well-being (i.e., work engagement and mental health), and that meaningful work influences strengths use through strengths knowledge. In our third study (Study 3), we intend to advance the theoretical understanding of the potential value and benefits of strengths knowledge and use in organizations by offering both: (a) empirical support for its positive relationships with work-related outcomes, such as meaningful work, work engagement and mental health, and (b) understanding the role of strengths knowledge and use as psychological mechanisms that explain how meaningful work is related to well-being. With this study, we seek to shed light on new pathways through which workers may find greater meaning in their work by cultivating knowledge and understanding of their own personal strengths. This may allow them to apply signature strengths and, in turn, experience vigor, dedication, and absorption in their jobs, as well as achieve higher levels of mental health, operationalized as the absence of severity of a mental problem over the past few weeks (Sánchez-López and Dresch, 2008). Our main contribution is to support the ability to be aware of and apply signature strengths as an effective and novel pathway to cultivate meaning and foster well-being and mental health at work. Investing in the development of inner resources at work is crucial to support workers to effectively cope with the effects of the Covid-19 crisis and achieve health and well-being during extraordinary times of material uncertainty and change (Salanova, 2020).

## 3. Study 1

Study 1 aims to analyze and validate the psychometric properties of the Spanish version of the SUS with a sample of Spanish and Latin American workers. Thus, we expect:

*H1*: The Spanish version of the SUS will demonstrate acceptable psychometric properties in terms of validity and reliability.

## 4. Materials and methods

### 4.1. Participants

A total of 617 workers from 11 private and public organizations in Spain (6 organizations; 57.5% of employees) and Latin America (5 organizations; Argentina = 15.6%; Mexico = 14.6%; Peru = 12.3%) participated in this study. Six companies belonged to the services sector (42.1% of employees), 3 to industry (38.2% of employees), 1 to public administration (11.7% of employees), and 1 to construction (7.9% of employees). Participants’ organizational tenure ranged from 0.6 to 48 years (*M* = 12.3; SD = 9.6). Respondents ranged in age from 19 to 66 years (18–24 age range = 6%; 25–34 age range = 22%; 35–44 age range = 35.7%; 45–54 = 24.2; > 54 = 12%); 52.4% were female, and 79.9% had an indefinite contract.

### 4.2. Instruments

#### 4.2.1. Strengths use scale

Strengths use was measured using the Spanish version of the original English version (Govindji and Linley, 2007). Respondents answered following the original instruction: *‘The following 14 questions ask about your strengths, that is, the things that you can do well or do best’.* All items were rated on a Likert scale ranging from 1 (strongly disagree) to 7 (strongly agree). The scale consists of 14 items (i.e., *‘I am regularly able to do what I do best’; ‘I am able to use my strengths in lots of different ways’*).

#### 4.2.2. Psychological capital

This construct was assessed by the Psychological Capital Questionnaire (PCQ-12; Avey et al., 2011), adapted from the PCQ-24 scale (Luthans et al., 2007). The scale consists of four dimensions: (1) self-efficacy, measured with three items (i.e., *“I am confident presenting information to a group of colleagues regarding this situation”*); (2) hope, measured with four items (i.e., *“If I should find myself in a jam trying to solve this situation, I could think of many ways to get out of it”*); (3) resilience, measured with three items (i.e., *“I take stressful things regarding this situation in stride”*); and (4) optimism, assessed by two items (i.e., *“I look on the bright side of things regarding this situation”*). Participants were asked to rate each of the statements using a 6-point Likert-type scale ranging from 0 (strongly disagree) to 5 (strongly agree).

#### 4.2.3. Happiness

This variable was assessed by the remembered well-being sub-scale from the Pemberton Happiness Index (PHI; Hervás and Vázquez, 2013). The scale contains 4 dimensions related to different domains of remembered well-being: (1) general well-being, measured with two items (i.e., “*I am very satisfied with my life*”); (2) eudaimonic well-being, measured with 6 items (i.e., “*My life is full of learning experiences and challenges that make me grow*”); (3) hedonic well-being, measured with two items (i.e., “*I enjoy a lot of little things every day*”); and (4) social well-being, measured with one item (i.e., “*I think I live in a society that lets me fully realize my potential*”). Participants were asked to rate each of the statements using a scale from 0 (fully disagree) to 10 (fully agree).

#### 4.2.4. Work engagement

Measured with the short 9-item version of the Utrecht Work Engagement Scale (UWES; Schaufeli et al., 2006). The scale includes three dimensions containing three items each: (1) vigor (i.e.: *“At my work, I feel bursting with energy”*); (2) dedication (i.e.: *“I am enthusiastic about my job”*); and (3) absorption (i.e.: *“I am immersed in my work”*). All the items were rated on a 7-point Likert scale ranging from 0 (*almost never*) to 6 (*almost always*).

#### 4.2.5. In–and extra-role performance

This variable was assessed by six items, included in the HEalthy and Resilient Organizations (HERO) questionnaire (Salanova et al., 2012), adapted from Goodman and Svyantek’s (1999) scale. Two dimensions (in-role performance and extra-role performance) were considered, with three items in each (i.e., “*I help when someone in the group is overworked*,” extra-role performance; “*I reach my goals at work*,” in role performance). Participants were asked to self-report each of the statements individually, using a Likert scale ranging from 0 (strongly disagree/never) to 6 (strongly agree/always).

### 4.3. Procedure

In accordance with scholars’ recommendations on scale translation (Muñiz et al., 2013), all scale items (*N* = 14) were translated from the original English version into Spanish by three independent Spanish native speakers with certified proficiency in English language skills. Next, one standardized version was developed by consensus. The items were then translated back into English by an English native professional translator. Both translations were compared and discrepancies were discussed between the research team until the final Spanish translation was accepted. Data were collected before the Covid-19 pandemic started, in the context of a broader research project about the role of leadership styles on employees’ work-related outcomes. After seeking permission from each participating organization’s CEO and reaching an agreement about the company’s participation, researchers conducted informational meetings about the project with middle managers. Spanish-speaking employees from Spain or Latin American countries, working in public or private organizations and having more than 6 months of tenure, were invited to participate in the research study. They were asked to collaborate in the investigation through meetings or memos, delivered by the directors of the company or members of the teams. The participants filled out an online questionnaire (including the Spanish version of the SUS) on a voluntary basis. The confidentiality of their replies was guaranteed according to GDPR laws, and informed consent was obtained from all individual participants before they started completing the questionnaire. The project was approved by the research ethics committee of the host university.

### 4.4. Data analyses

All statistical analyzes were conducted using IBM SPSS 26 (IBM Corp, 2019), SPSS AMOS 23 (Arbuckle, 2014), and JASP computer software (JASP Team, 2020). The same analytical procedure was used for both Study 1 and Study 2.

First, we examined descriptive statistics including means, standard deviations, skewness, kurtosis, and Pearson’s coefficients for all the variables, included in the study. To explore internal consistency, we calculated Cronbach’s Alpha and McDonald’s Omega (Peters, 2014). We explored gender and country differences as well, using Student’s *t-*test (Field, 2009). To determine the number of factors to extract we conducted an exploratory factor analysis (EFA) following the maximum likelihood estimation approach, and parallel analysis. To examine the factor structure of the SUS, we conducted confirmatory factor analysis (CFA) utilizing the maximum likelihood estimation approach. To establish goodness-of-fit of the data to the proposed structures, we computed the chi–square (*χ*^2^) and normed *χ*^2^, root–mean–squared error of approximation comparative fit index (CFI), (RMSEA) with a confidence interval (90% CI), and standardized root mean residual (SRMR). We followed model fit guidelines and cut-off points, published by the European Journal of Psychological Assessment (Schweizer, 2010). To explore invariance, we followed the steps, indicated by Putnick and Bornstein (2016), and tested for configural, metric, and scalar invariance by gender and country. To evaluate the changes in fit, we used absolute differences in CFI or SRMR, where increases of less than 0.01 are equivalent (Cheung and Rensvold, 2002).

## 5. Results

Descriptive statistics, reliability coefficients, and Pearson’s correlations for all variables, included in the study are presented in Table 1. Internal consistency requirement of >0.70 for all variables is confirmed in both Cronbach’s Alpha and McDonald’s Omega coefficients. Furthermore, all correlations between strengths use and well-being (i.e., psychological capital, happiness, and work engagement), and performance (i.e., in-and extra-role performance) variables are significant (*p* < 0.001) and positive.

**Table 1:** Descriptive statistics, reliability coefficients, and Pearson’s correlations for all study 1 variables.

	Mean (SD)	*α*	*ω*	1	2	3	4	5
1.Strength Use	5.00(0.71)	0.95	0.95					
2.Psychological Capital	4.85(0.70)	0.89	0.88	0.719^**^				
3.Happiness	5.09(0.60)	0.87	0.87	0.650^**^	0.689^**^			
4.Work Engagement	4.89(0.81)	0.91	0.91	0.655^**^	0.590^**^	0.562^**^		
5.In Role Performance	5.19(0.71)	0.80	0.80	0.602^**^	0.600^**^	0.499^**^	0.422^**^	
6.Extra Role Performance	5.28(0.71)	0.89	0.88	0.498^**^	0.444^**^	0.380^**^	0.400^**^	0.591^**^

∗∗*p* < 0.001.

T-test results indicated significant differences when comparing participants from Spain against Latin-American countries, *t* (612) = −5.633, *p* < 0.001, *d* = −0.455, 95% IC (−0.615, −0.294). No significant differences were detected between male and female participants, *t* (611) = −0.975, *p* = 0.330, *d* = −0.079, 95% IC (−0.237, −0.080).

The total skewness and kurtosis values were − 0.644 and 0.599 respectively, which indicates the assumption of normality in the distribution is not violated (Field, 2009). However, upon closer inspection, skewness and kurtosis values exceeded the suggested threshold for several individual items in the Strengths Use Scale presented in Table 2. Following Field (2009) we computed the Kaiser-Meyer-Olkin measure for sampling adequacy (KMO = 0.946) and Bartlett’s test of sphericity (*p* < 0.001). Both tests proved the adequacy of the data for further analyzes. Furthermore, EFA indicated a single factor explained 59.4% with items’ factor loadings between 0.64 and 0.86 (see Table 2). The first factor had an eigenvalue of 8.73, with the remaining factors clearly below the intersection line (0.702 to 0.176).

**Table 2:** Descriptive statistics and factor loadings of the strength use scale (Study 1).

Items (1 ‘strongly disagree’ → 7 ‘strongly agree’)	*M* (SD)	*S*	*K*	Factor Loadings	*α* index if item is dropped	*ω* index if item is dropped
(1) I am regularly able to do what I do best.	5.13 (0.86)	−1.06	1.834	0.71**	0.948	0.948
(2) I always play to my strengths.	5.05 (0.86)	−0.969	1.858	0.73**	0.948	0.947
(3) I always try to use my strengths.	5.18 (0.83)	−1.04	2.201	0.78**	0.947	0.946
(4) I achieve what I want by using my strengths.	5.13 (0.80)	−0.676	0.161	0.80**	0.946	0.946
(5) I use my strengths every day.	5.02 (0.89)	−0.731	0.321	0.81**	0.946	0.946
(6) I use my strengths to get what I want out of life.	5.16 (0.82)	−0.715	0.084	0.83**	0.946	0.946
(7) My work gives me lots of opportunities to use my strengths.	5.16 (0.80)	−0.708	0.085	0.86**	0.945	0.945
(8) My life presents me with lots of different ways to use my strengths.	4.85 (1.09)	−1.274	2.533	0.65**	0.950	0.949
(9) Using my strengths comes naturally to me.	4.83 (1.06)	−1.207	2.194	0.64**	0.950	0.949
(10) I find it easy to use my strengths in the things I do.	5.08 (0.90)	−1.267	3.351	0.80**	0.946	0.945
(11) I am able to use my strengths in lots of different situations.	4.97 (0.92)	−1.247	3.717	0.79**	0.946	0.946
(12) Most of my time is spent doing the things that I am good at doing.	4.66 (1.05)	−0.824	1.121	0.66**	0.950	0.950
(13) Using my strengths is something I am familiar with.	4.97 (0.90)	−0.784	1.093	0.83**	0.946	0.945
(14) I am able to use my strengths in lots of different ways.	4.90 (0.92)	−0.599	0.124	0.79**	0.946	0.946

∗∗*p* < 0.001; SD, standard deviation; S, skewness; K, kurtosis.

**Figure 1:**
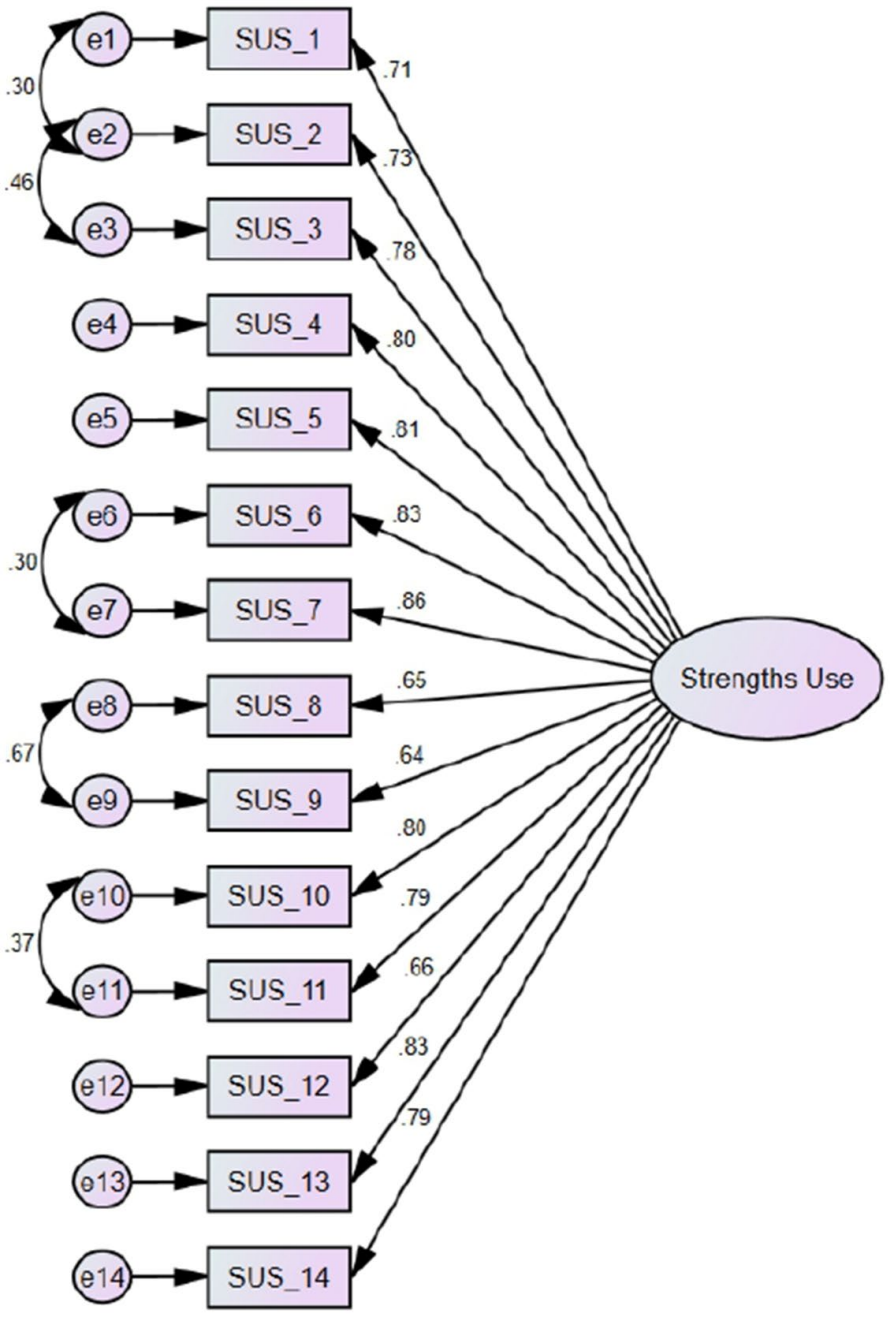
Factorial Structure of the Spanish version of the strengths Use scale (Study 1).

Results for the CFA and invariance analyzes are presented in Table 3. The initial single factor, model tested in the CFA (M1) analyzes showed a less than satisfactory fit to the data. Thus, modification indexes were calculated to examine possible opportunities to correlate error terms. Upon the introduction of five error term correlations, adequate fit to the data was achieved in M2. The error terms (i.e., e1↔e2; e2↔e3: e6↔e7; e8↔e9, e10↔e11) can be attributed to similar wording and content. Moreover, item error terms were allowed to covary, since all items presented acceptable factor loading to the single factor solution. The final factorial structure of the Spanish version of the Strength Use Scale is presented in Figure 1.

**Table 3:** Fit indexes for single group and multi-group confirmatory factor analyzes for the strength use scale (Study 1).

	*χ* ^2^	df	*χ*^2^/df	RMSEA	90% CI	CFI	TLI	SRMR	CMs	∆ *χ*^2^(∆ df)	∆ CFI	∆ SRMR
Single group CFA												
M1 Single Factor	1045.119**	77	13.573	0.143	[0.136, 0.151]	0.862	0.837	0.059	-	-	-	-
M2 Single Factor revised	333.390**	72	4.630	0.077	[0.069, 0.085]	0.963	0.945	0.034	M1-M2	711.729 (5)**	0.101	0.025
Multiple Group (Country)												
M3 configural invariance	479.993**	144	3.333	0.088	[0.080, 0.097]	0.951	0.938	0.040	-	-	-	-
M4 metric invariance	508.402**	157	3.238	0.087	[0.078, 0.095]	0.948	0.940	0.060	M3-M4	28.409 (13)ns	0.003	0.020
M5 scalar invariance	558.125**	170	3.283	0.087	[0.079, 0.096]	0.943	0.939	0.061	M4-M5	49.723 (13)ns	0.005	0.001

∗∗*p* < 0.001; *χ*^2^, Chi-square; df, degree of freedom; RMSEA, root mean square error of approximation; CI, 90% confidence interval; CFI, comparative fit index; IFI, incremental fit index; SRMR, standardized root mean square residual; CMs, comparisons between models.

Next, based on the t-test results, we conducted invariance analysis comparing the stability of the factor structure of the Spanish version of the Strengths Use Scale between participants from Spain and Spanish-speaking Latin American countries (i.e., Argentina, Chile, Uruguay, Colombia, Ecuador, Mexico, and Costa Rica). Results are shown in Table 3.

The baseline model (M3) showed adequate fit, supporting configural invariance. Next, constraints were imposed on all factor loadings, making them equal to examine metric invariance. The resulting model also showed adequate fit indexes (see M4). Comparison between M3 and M4 yielded a non-significant chi-squared difference test. As for the CFI and SRMR indexes, the differences were below the 0.01 threshold for CFI and above for SRMR, thus metric invariance was only partially supported. Finally, we imposed equality constraints on all intercepts to examine scalar invariance. This model (M5) showed acceptable fit indexes. Comparison between M4 and M5 yielded a non-significant chi-squared test difference, and no differences in the CFI and SRMR indexes. Considering the present results, we can say that the factorial structure of the Spanish version of the Strengths Use Scale remains relatively stable across Spanish-speaking Latin-American countries although strict invariance was not achieved.

## 6. Brief discussion

In this study, we used a combination of exploratory and confirmatory statistical approaches to validate the Strengths Use Scale. The results confirmed the structure of the scale measuring a single strength use factor, developed in previous research (Govindji and Linley, 2007). Internal consistency and reliability were established, as well as convergent validity. All well-being (i.e., work engagement, happiness, and psychological capital) and performance (i.e., in-and extra-role performance) measures correlated with strengths use in the expected directions. After allowing error terms of a few items to covary due to similar wording (e1↔e2; e2↔e3), or a context view when seeking opportunities to apply strengths (e6↔e7; e8↔e9, e10↔e11), the SUS scale showed satisfactory fit indexes. Our findings suggest that the Spanish version of the SUS scale is equivalent to the original English version, and represents a reliable and valid measure of strengths use. The original item pool was conserved, and all items showed acceptable factor loadings. Additionally, the SUS maintains its factor structure across Latin American Spanish speaking countries with stability.

## 7. Study 2

This study aims to analyze and validate the psychometric properties of the Spanish version of the SKS with a sample of Spanish and Latin American workers. Thus, we expect:

*H2*: The Spanish version of the SKS will demonstrate acceptable psychometric properties in terms of validity and reliability.

## 8. Materials and methods

### 8.1. Participants

A total of 365 workers from a wide variety of professions participated in this study (24.6% from the education sector, 18% from the industrial sector, and 16,3% from the services sector, among others). Participants were either from Spain (*N* = 208; 57%) or Spanish speaking Latin American Countries (*N* = 157, 43%). Participants from Latin American Countries were from Argentina (*N* = 68), Uruguay (*N* = 39), Chile (*N* = 21), Mexico (*N* = 20), and Colombia (*N* = 9). Participant’s age ranged from 18 to 72 years (*M* = 41.62, SD = 10.27), and 53% were female. It is important to note that the present sample is independent from the one, utilized for Study 1.

### 8.2. Instruments

#### 8.2.1. Strengths knowledge scale

Strengths knowledge was measured using the Spanish version of the original English SKS version (Govindji and Linley, 2007). The scale contains 8 items (i.e., “*I know when I am at my best*”) and exhibited a high internal consistency in the original validation. All items were rated on a 7-point Likert scale, ranging from strongly disagree to strongly agree.

#### 8.2.2. Mental health

This variable was measured with the Spanish validation (Sánchez-López and Dresch, 2008) of the 12-item General Health Questionnaire (GHQ-12; Goldberg and Williams, 2000). The scale consists of 12 items, assessing the severity of a mental problem over the past few weeks, using a 4-point Likert scale from 0 to 3. The score was used to generate a total score ranging from 0 to 36. The positive items were corrected from 0 (always) to 3 (never) and the negative ones from 3 (always) to 0 (never). High scores indicate worse health.

#### 8.2.3. Work engagement

Participants completed the UWES (Schaufeli et al., 2006), described in Study 1. *α* = 0.88.

#### 8.2.4. In-role and extra-role performance

This variable was assessed by two items, included in the HEalthy and Resilient Organizations (HERO) questionnaire (Salanova et al., 2012), adapted from Goodman and Svyantek’s (1999) scale. Two dimensions (in-role performance and extra-role performance) were considered, with one item each (i.e., “*I help when someone in the group is overworked*”; extra-role performance). Participants were asked to self-report each of the statements individually, using a Likert scale ranging from 0 (strongly disagree/never) to 6 (strongly agree/always).

### 8.3. Procedure

All scale items (*N* = 8) for the SKS followed the same translation and re-translation procedure described in Study 1.

Data were collected in the context of a broader research project about differences on well-being and work-related variables between teleworkers versus office-based workers during the first COVID-19 pandemic outbreak of 2020. The same inclusion criteria (Spain or Latin American country; Spanish-speaking language; employees working in public or private organizations; minimum of 6 months tenure) for participating in the research study, indicated in Study 1, were used for Study 2. Respondents were recruited through an online questionnaire via Qualtrics. The link to the questionnaire was available on the authors’ research team’s website and disseminated via social networks. The participants filled out an online questionnaire (including the Spanish version of the SKS) on a voluntary basis. The confidentiality of their replies was guaranteed according to GDPR laws, and informed consent was obtained from all individual participants before they started completing the questionnaire. The project was approved by the research ethics committee of the host university.

### 8.4. Data analyses

The same statistical procedures, detailed in Study 1 were used for Study 2.

## 9. Results

Descriptive statistics, reliability coefficients, and Pearson’s correlations for all variables, included in the study, are presented in Table 4. Internal consistency requirement of >0.70 in both Cronbach’s Alpha and McDonald’s Omega coefficients for all measurement instruments is confirmed, except for in-role and extra-role performance, which were single-item measures. Pearson’s correlations between the SKS and work engagement, meaningful work, and performance were significant (*p* < 0.001) and positive, whereas the correlation with General Health (GHQ) was significant (*p* < 0.001) and negative.

**Table 4:** Descriptive statistics, reliability coefficients, and Pearson’s correlations for all study 2 variables.

	Mean (SD)	*α*	*ω*	1	2	3	4	5
1. Strength Knowledge	4.40(0.79)	0.90	0.90					
2. Meaningful Work	5.36(0.91)	0.91	0.87	0.490^**^				
3. Mental Health	2.70(1.01)	0.90	0.90	−0.320^**^	−0.185^**^			
4. Work Engagement	3.89(1.17)	0.88	0.88	0.402^**^	0.432^**^	−0.562^**^		
5. In Role Performance	4.22(1.13)	-	-	0.316^**^	0.181^**^	−0.407^**^	0.536^**^	
6. Extra Role Performance	5.06(1.14)	-	-	0.298^**^	0.263^**^	−0.310^**^	0.366^**^	0.425^**^

∗∗*p* < 0.001.

The *t*-test results showed significant differences in the study variables when comparing participants from Spain against those from Latin-American countries, *t* (456) = −2.240, *p* < 0.05, *d* = −0.239, 95% IC (−0.449, −0.029). No significant differences were detected between male and female participants, *t* (353) = −1.283, *p* = 0.200, *d* = −0.147, 95% IC (−0.371, 0.078).

The total skewness and kurtosis values were − 0.362 and 1.479 respectively, which indicates the assumption of normality in the distribution is violated (Field, 2009). Specifically, kurtosis values higher than 1 indicate the presence of outliers (Field, 2009). Upon inspection of the data distribution through a histogram plot, the presence of outliers was confirmed at the negative end of the distribution, that is two standard deviations below the mean. Closer inspection skewness and kurtosis values for each of the Strengths Knowledge Scale items shows the same pattern of deviation. Results are shown in detail in Table 5.

**Table 5:** Descriptive statistics and factor loadings of the strengths knowledge scale (study 2).

Items (1 ‘strongly disagree’ → 7 ‘strongly agree’)	M (SD)	*S*	*K*	Factor Loadings	α index if item is dropped	ω index if item is dropped
(1) Other people see the strengths that I have	4.42 (0.96)	−0.733	2.546	0.50**	0.904	0.909
(2) I have to think hard about what my strengths are	3.45 (1.44)	−0.322	−0.211	0.35**	0.928	0.935
(3) I know what I do best	4.59 (0.96)	−0.711	1.853	0.87**	0.867	0.878
(4) I am aware of my strengths	4.52 (0.99)	−0.526	1.102	0.91**	0.864	0.873
(5) I know the things I am good at doing	4.59 (0.96)	−0.633	1.646	0.92**	0.867	0.877
(6) I know my strengths well	4.47 (0.98)	−0.468	1.302	0.92**	0.863	0.873
(7) I know the things I do best	4.55 (0.90)	−0.300	1.304	0.91**	0.867	0.876
(8) I know when I am at my best	4.66 (0.99)	−0.569	0.922	0.68**	0.885	0.895

∗∗*p* < 0.001; SD, standard deviation; S, skewness; K, kurtosis.

Despite the violation of the assumption of normality, following Field (2009) we computed the Kaiser-Meyer-Olkin measure for sampling adequacy (KMO = 0.920) and Bartlett’s test of sphericity (*p* < 0.001). Both tests proved the adequacy of the data for further analyzes. The EFA indicated a single factor explained 62.1% of the extracted variance, with item factor loadings between 0.48 and 0.91 (see Table 5). The first factor had an eigenvalue of 5.23, with the remaining factors clearly below the intersection line (0.896 to 0.105). Items 1 and 2 showed relatively low factor loadings but, considering the overall psychometric properties of the scale, they remained acceptable when included (*α* = 0.90 and *ω* = 0.90). Therefore, we decided to retain them for further analyzes and conserve the original structure of the strengths knowledge scale.

**Figure 2:**
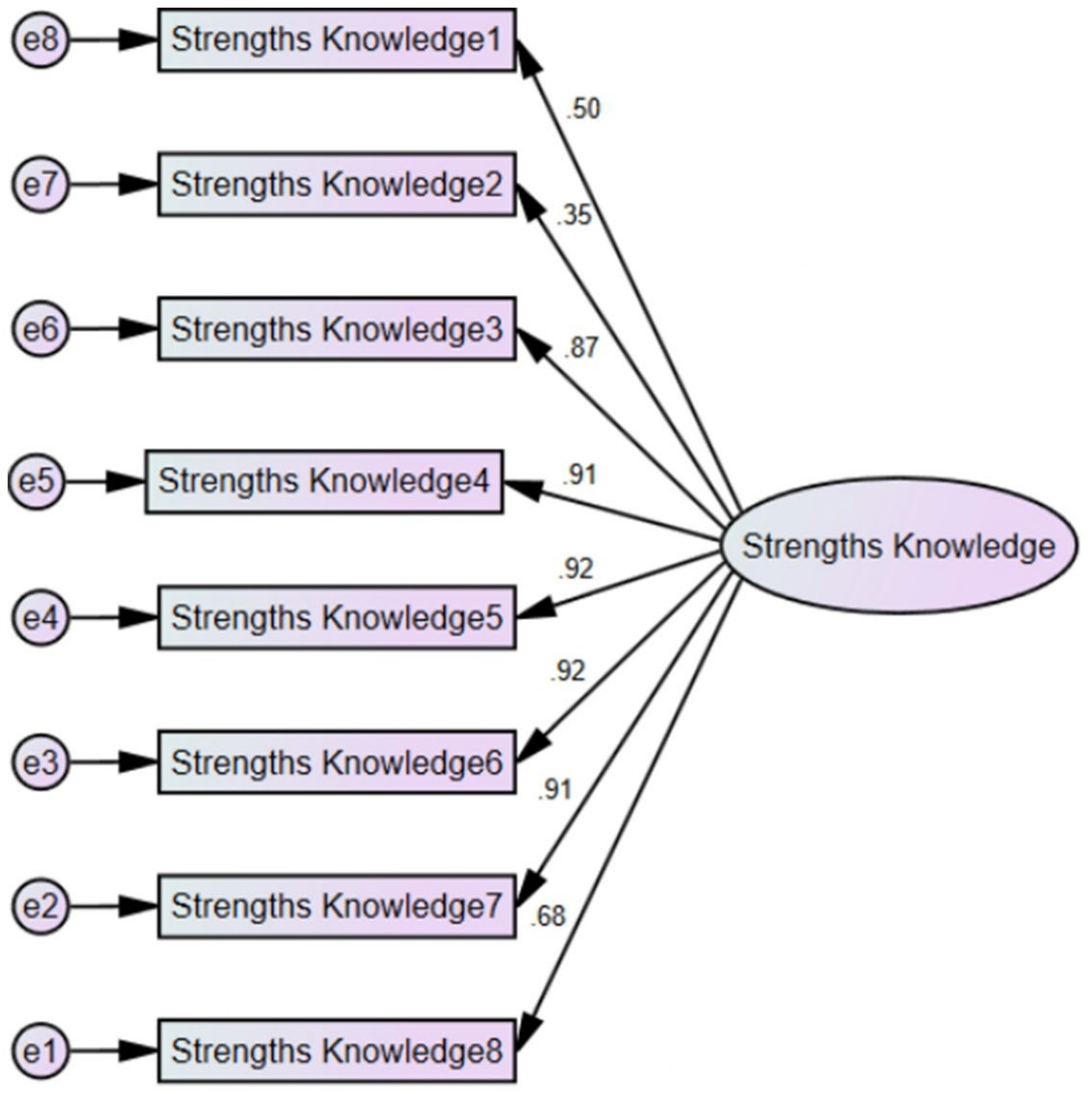
Factorial Structure of the Spanish version of the strengths Knowledge scale (study 2).

Results for the CFA and invariance analyzes are presented in Table 6. The initial single factor model, tested in the CFA (M1) analyzes, showed satisfactory fit to the data. To confirm our initial decision to conserve items 1 and 2, we tested for an additional model, excluding both items (M2). Fit indexes did not increase significantly, thus we opted to conserve items 1 and 2 and keep the original item pool. The final factorial structure of the Spanish version of the Strength Knowledge Scale is presented in Figure 2.

**Table 6:** Fit indexes for single group and multi-group confirmatory factor analyzes for the strengths knowledge scale (study 2).

	*χ* ^2^	df	*χ*^2^/df	RMSEA	90% CI	CFI	TLI	SRMR	CMs	∆ *χ*^2^(∆ df)	∆ CFI	∆ SRMR
Single Group CFA												
M1 Single Factor	183.990**	20	9.699	0.103	[0.089, 0.116]	0.969	0.957	0.027	-	-	-	-
M2 Single Factor 6 items	162.575**	15	10.838	0.117	[0.101, 0.133]	0.970	0.957	0.027	M1-M3	21.215 (15)ns	0.001	0.000
Multiple Group (Country)												
M3 configural invariance	212.545**	40	5.313	0.105	[0.092, 0.119]	0.967	0.954	0.031	-	-	-	-
M4 metric invariance	226.152**	47	4.811	0.099	[0.086, 0.112]	0.966	0.959	0.049	M2-M3	13.607 (17)ns	0.001	0.018
M5 scalar invariance	230.296**	54	4.264	0.092	[0.080, 0.104]	0.967	0.965	0.045	M3-M4	4.144 (17)ns	0.001	0.004

∗∗*p* < 0.001; *χ*^2^, Chi-square; df, degree of freedom; RMSEA, root mean square error of approximation; CI, 90% confidence interval; CFI, comparative fit index; IFI, incremental fit index; SRMR, standardized root mean square residual; CMs, comparisons between models.

Next, based on the t-test results, we conducted invariance analysis, comparing the stability of the factor structure of the Spanish version of the strengths knowledge scale between participants from Spain and those from Spanish-speaking Latin American countries (i.e., Argentina, Chile, Uruguay, Colombia, and Mexico). Results are shown in Table 6. The baseline model (M3) showed adequate fit, supporting configural invariance. Next, constraints were imposed on all factor loadings, making them equal to examine metric invariance. The resulting model also showed adequate fit indexes (see M4). Comparison between M3 and M4 yielded a non-significant chi-squared difference test. As for the CFI and SRMR indexes, the differences were below the 0.01 threshold for CFI, and slightly above for the SRMR, thus metric invariance was only partially supported. Finally, equality constraints were imposed on all intercepts to examine scalar invariance. This model (M5) also showed acceptable fit indexes. Comparison between M4 and M5 yielded a non-significant chi-squared test difference and minimal differences below the 0.01 threshold for CFI and SRMR indexes. Considering the present results, we can say that the factorial structure of the Spanish version of the strengths knowledge scale remains relatively stable across Spanish-speaking Latin-American countries although of all the three levels of invariance that were tested, metric invariance was only partially supported.

## 10. Brief discussion

In this study, we used a combination of exploratory and confirmatory statistical approaches to validate the Strength Knowledge Scale. The results confirmed the structure of the scale, measuring a single strengths knowledge factor, developed in previous research (Govindji and Linley, 2007). Internal consistency and reliability were established, as well as convergent validity. All well-being (i.e., work engagement and mental health) and performance (i.e., in-and extra-role performance) measures correlated with strengths knowledge in the expected directions. Our findings suggest that the Spanish version of the SKS scale is equivalent to the original English version, and represents a reliable and valid measure of strengths knowledge. Despite the lower than desirable factor loadings, the original item pool was conserved, since excluding them only resulted innon-significant changes in model fit, and no changes in reliability were observed. Additionally, the SKS maintains its factor structure across Latin American Spanish speaking countries with stability.

## 11. Study 3

Study 3 aims to analyze the mediating roles of strengths knowledge and strengths use in the link between meaningful work, work engagement, and mental health. The hypothesized models were explored through the following hypotheses:

*H3*: Meaningful work is indirectly associated with strengths use through the partial mediating role of strengths knowledge.

*H4*: Meaningful work is indirectly associated with work engagement through the partial mediating role of strengths use.

*H5*: Meaningful work is indirectly associated with mental health through the partial mediating role of strengths use.

## 12. Materials and methods

### 12.1. Participants

Convenience sampling yielded 798 workers from organizations of different sectors (42% from the services sector, 28% from the education sector, 19% from the industrial sector, and 11% from the healthcare sector). This sample was independent from Study 1 and Study 2. Forty-four percent of the participants were from Spain, and 56% from Latin American countries. Participants ranged in age from 18 to 71 years (*M* = 42.03, SD = 10.57), and 58% were female.

### 12.2. Instruments

The Spanish validated versions of the strengths knowledge scale (8-items) and SUS (short 4-items version), meaningful work, UWES work engagement and GHQ-12, all described in Study 1 and/or Study 2, were used for Study 3.

### 12.3. Procedure

For data collection, we followed the same procedure as in Study 2.

### 12.4. Data analyses

First, descriptive analyzes (e.g., means, standard deviations, and Cronbach’s alpha coefficients) were calculated, in addition to the bivariate correlations between all the variables, using the IBM SPSS Statistics 25.0 package. Second, structural equation modeling (SEM) was applied to test the structural relations in the hypothesized models using AMOS. The maximum likelihood method was used, and goodness-of-fit of each model was determined by considering absolute and relative indexes (Schermelleh-Engel et al., 2003): *χ*^2^, *χ*^2^/df, incremental fit index (IFI), CFI, normed fit index (NFI), RMSEA, standardized root-mean-square residual (SRMR), and Akaike information criterion (AIC). Finally, the product of coefficients method (MacKinnon et al., 2002) was applied to test the mediation hypotheses.

## 13. Results

### 13.1. Preliminary analyzes

Table 7 shows means, standard deviations, Cronbach’s α indexes, and Pearson’s correlations among the study variables. As expected, the internal consistency of all the measures was satisfactory, and all the inter-correlations among the study variables were significant (*M* = 0.507), ranging from 0.186 to 0.786 (*p* < 0.01).

**Table 7:** Descriptive statistics, reliability coefficients, and Pearson’s correlations for all study 3 variables.

	Mean (SD)	*α*	*ω*	1	2	3	4
1. Strength knowledge	4.38(0.76)	0.90	0.90				
2. Strength use	4.26(0.88)	0.88	0.89	0.786^**^			
3. Meaningful work	5.23(0.88)	0.90	0.91	0.470^**^	0.515^**^		
4. Mental health	2.60(0.99)	0.89	0.89	−0.443^**^	−0.462^**^	−0.186^**^	
5. Work engagement	3.97(1.11)	0.86	0.86	0.367^**^	0.414^**^	0.435^**^	−0.538^**^

∗∗*p* < 0.001.

### 13.2. Model fit: structural equation modeling

**Figure 3:**
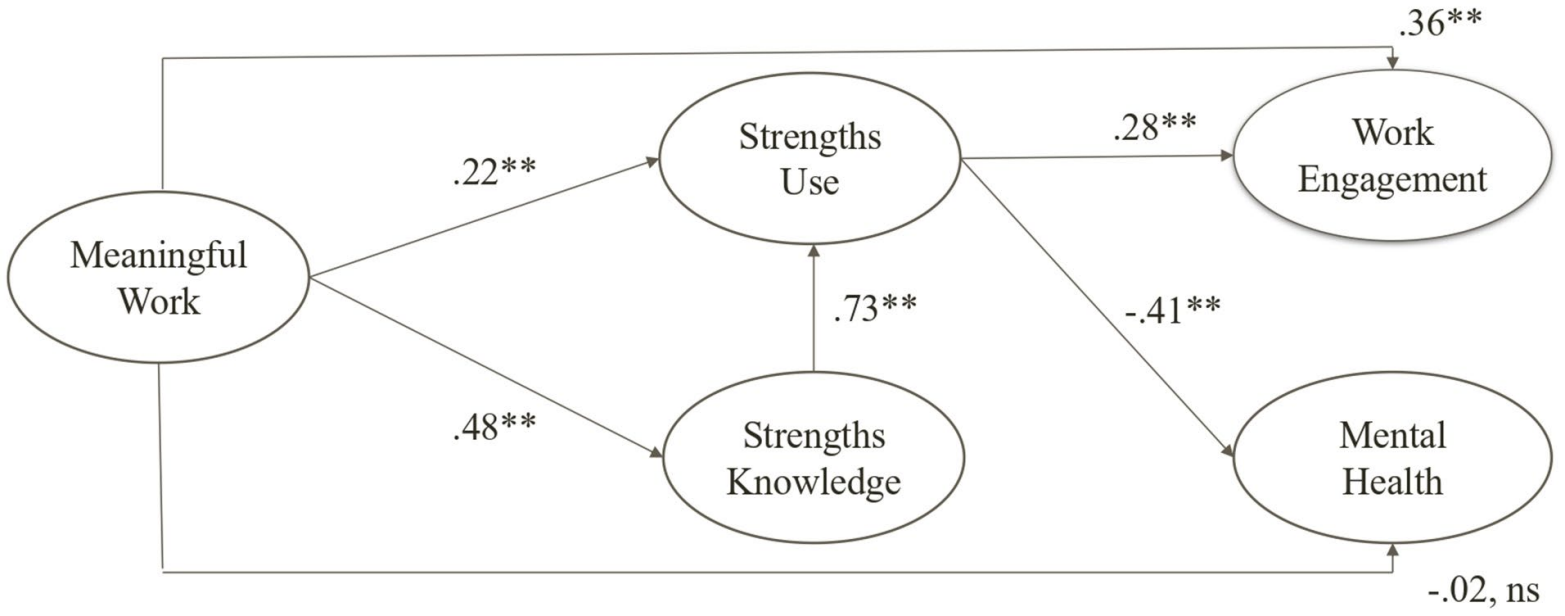
The final model (M4) with standardized path coefficients (Study 3). Mental health is operationalized in the model as the absence of severity of a mental problem. Thus, the path from antecedent and mediating variable to this construct is negative in the figure.

Meaningful work, strengths knowledge, strengths use, work engagement, and mental health are represented as latent variables in the structural model, shown in Figure 3. Following James et al. (2006), four models were tested to verify the hypotheses. Model 1 (M1) assumes that strengths knowledge fully mediates the relationship between meaningful work and strengths use. The results, represented in Table 8, show that M1 presents a good fit to the data, and that all the fit indices met the criteria. The path from meaningful work to strengths knowledge was positive and statistically significant (*β* = 0.49, *p* < 0.001), as was the path from strengths knowledge to strengths use (*β* = 0.84, *p* < 0.001).

**Table 8:** Fit indices of the structural equation models for study 3.

Model	*χ* ^2^	*p*	*d.f.*	CFI	TLI	RMSEA	Comparison	*χ*^2^ Diff	*p*
M1	937.558	0.000	199	0.943	0.934	0.068			
M2	882.802	0.000	198	0.947	0.939	0.066	M1-M2	54.756	<0.001
M3	2221.646	0.000	606	0.919	0.911	0.058			
M4	2157.705	0.000	604	0.922	0.914	0.057	M3-M4	63.941	<0.001

M1 = Model 1; M2 = Model 2; M3 = Model 3; M4 = Model.

Next, a new model (M2; our research model) was developed, which assumes that strengths knowledge plays a partial mediating role between meaningful work and strengths use. In other words, there is also a direct relationship between meaningful work and strengths use. The results indicate that M2 also presents a good fit to the data, and that all the fit indices met the criteria. The path from meaningful work to strengths knowledge was positive and statistically significant (*β* = 0.48, *p* < 0.001), as was the path from strengths knowledge to strengths use (*β* = 0.72, *p* < 0.001). As shown in Table 8, M2 revealed a better fit to the data, with statistically significant differences between the two models. Thus, we opted for our research model (M2), which assumes that meaningful work is indirectly associated with strengths use through the partial mediating role of strengths knowledge.

According to MacKinnon et al. (2002), the product of coefficients method was estimated to test the mediation hypothesis (H3). The mediated effect of strengths knowledge in the relationship between meaningful work and strengths use was statistically significant (*P* = Ζ_α_ · Ζ_β_ = 380.2, *p* < 0.001). Additionally, the direct relationship between meaningful work and strengths use (*β* = 0.22, *p* < 0.001) was also statistically significant. These results suggest a partial mediation effect of strengths knowledge between meaningful work and strengths use, supporting our H3.

To test our H4 and H5, a new Model (M3) was developed, which assumes that strengths use fully mediates the relationship between meaningful work and work engagement (H4), and mental health (H5). Results indicate a good fit to the data, and that all the fit indices met the criteria. The paths from meaningful work to strengths use (*β* = 0.26, *p* < 0.001) was positive and statistically significant, as was the path from strengths use to work engagement (*β* = 0.52, *p* < 0.001), and from strengths use to mental health (*β* = −0.43, *p* < 0.001). Finally, we compared M3 with M4, which assumes that strengths use partially mediates the relationship between meaningful work and work engagement (H4), and also between meaningful work and mental health (H5). In other words, there are also direct relationships between meaningful work and work engagement, and between meaningful work and mental health. The results indicate that M4 also presents a good fit to the data, and that all the fit indices met the criteria. The paths from meaningful work to strengths use (*β* = 0.22, *p* < 0.001) was positive and statistically significant, as was the path from strengths use to work engagement (*β* = 0.28 *p* < 0.001), from strengths use to mental health (*β* = −0.41, *p* < 0.001), and from meaningful work to work engagement (*β* = 0.36 *p* < 0.001). As shown in Table 8, M4 revealed a better fit to the data, with statistically significant differences between the two models. Thus, we opted for M4, which assumes that meaningful work is indirectly associated with work engagement and with mental health through the partial mediating role of strengths use.

To assess the mediation effects for H4 and H5, the MacKinnon test was estimated. The mediating effects of strengths use in the link between meaningful work and work engagement (*P* = Ζ_α_ · Ζ_β_ = 116.2, *p* < 0.001), and between meaningful work and mental health (*P* = Ζ_α_ · Ζ_β_ = 138.1, *p* < 0.001) were statistically significant, thus confirming the mediation effects. However, while the link from meaningful work to work engagement was positive and significant (supporting H4), the link to mental health (*β* = −0.02, *ns*) was not, thus partially supporting H5.

## 14. Brief discussion

Results from Study 3 fully supported H3, indicating a partial mediating role of strengths knowledge in the link between meaningful work and strengths use. Moreover, while H4 was supported, suggesting a partially mediating role of strengths use in the relationship between meaningful work and work engagement, H5 was partially supported, suggesting a fully mediating role of strengths use between meaningful work and mental health.

## 15. General discussion

The purpose of the current study was twofold: first, to validate and adapt the Strengths Use (Study 1) and Strengths Knowledge (Study 2) Scales to the Spanish and Latin American context; and second, to explore the mediating roles of strengths use and strengths knowledge between meaningful work and well-being (i.e., work engagement and mental health).

In the case of Studies 1 and 2, using a combination of exploratory and confirmatory statistical approaches, results indicated that the Spanish SUS and SKS were adequate instruments with good psychometric properties. Findings confirmed the structure of each scale, measuring a single factor, developed in previous research (Govindji and Linley, 2007). The adequate levels of reliability and validity are sufficient to support the use of the scales and the interpretation of the scores in Spanish and Latin American working populations, equivalent to the study samples. Internal consistency, reliability, and convergent validity were tested. Regarding criterion validity, work engagement, happiness, psychological capital, and performance measures, tested in Study 1, correlated with the SUS as hypothesized, as well as work engagement, mental health and in-role and extra-role performance measures, tested in Study 2, correlated with the SKS. Additionally, the scales showed satisfactory fit indexes. Overall, findings from this study suggest that the Spanish versions of the SUS and SKS are equivalent to the original English versions, and represent reliable and valid measures of strengths use and strengths knowledge. The original item pools were conserved, and all items showed acceptable factor loadings. Moreover, scales invariances were also demonstrated, maintaining their factor structure across Latin American Spanish speaking countries. Thus, our findings fully support H1 and H2.

Studies 1 and 2 replicated the hypothesized relationships between strengths use and strengths knowledge, and several measures of well-being, such as mental health (Duan et al., 2019) and subjective and psychological well-being (Niemiec, 2019). Specifically, these studies contribute to the strengths approach by confirming the value of strength awareness and application in fostering positive outcomes, such as work engagement (Harzer and Ruch, 2013; Bakker and Van Wingerden, 2021) and job performance (Huber et al., 2020) in the organizational context. These results contribute to the JD-R model’s theoretical framework by supporting the role of personal strengths as personal resources that may foster feelings of excitement, happiness, and optimism, leading to a motivational process from which work engagement and performance arise (Harzer, 2020).

Regarding Study 3, interesting results emerged that should be mentioned. First, findings confirmed the positive and direct links between meaningful work and strengths use, and between meaningful work and strengths knowledge. In addition, strengths knowledge played a partial mediating role between meaningful work and strengths use, thus confirming H3. This result revealed that meaningful work has an influence on strengths use through strengths knowledge. In other words, employees who perceive their work as meaningful are increasingly capable of discovering and using their character strengths at work. In this sense, meaningful work facilitates the process of becoming aware, exploring, and utilizing personal strengths at work. Meaning influences the motivational process of engagement by creating the space for strengths to be recognized, acknowledged, and integrated through reflections around personal growth, contributions to the greater good, and crafting a positive sense from a personal perspective. This result reinforces the theoretical notion that employees are intrinsically motivated to do what they do best when they are aware and know their strengths well (Duan et al., 2019). Thus, we contribute to the meaningful work literature (Steger, 2019) by confirming that perceiving work as meaningful is a vital job resource that provides the opportunity to discover and get insight into one’s personal strengths and, in turn, be motivated and more confident to use them.

Results from this study also confirmed the partial mediating role of strengths use in the relationship between meaningful work and work engagement, and a fully mediating role of strengths use in the relationship between meaningful work and mental health. These findings highlight the ability to use and apply personal strengths as an underlying mechanism that explains how meaningful work exerts its influence on work engagement and mental health. Previous research has revealed that one of the consequences of sensing work as significant, positive, and meaningful, is the ability to use one’s strengths at work (Arnold et al., 2007; Steger et al., 2013). This suggests an interactive effect in which meaningful work acts as a vital job resource that facilitates the opportunity to discover and use character strengths. In turn, acquiring knowledge about personal strengths and intentionally putting them to use offers workers a practical pathway to implement meaningful actions within the frame of everyday work. Simply put, the combination of strengths knowledge and strengths use offers a concrete approach to translating meaning from ideas into tangible actions.

Furthermore, a positive and direct link was also found between meaningful work and work engagement, indicating that perceiving work as meaningful facilitates employees’ abilities to invest themselves in their work and be more vigorous, dedicated and absorbed in the job. This result is in line with previous research that demonstrated the predictive value of meaningful work on work engagement (Steger et al., 2013; Allan et al., 2019). In this sense, having a clear sense of purpose and meaning makes it easier for workers to invest energy and focus on a path that resonates with their deepest aspirations and transcendent desires.

On the other hand, the direct link between meaningful work and mental health was not significant, thus suggesting that perceiving work as meaningful has benefits for mental health through the use of strengths as an underlying mechanism. This particular result offers a more nuanced description of how meaning exerts an effect on mental health and on the capacity to cope with stressors and negative emotions. Essentially, our results suggest that the presence of meaning by itself is not sufficient to gain a significant positive effect on mental health. It seems meaningful work requires a clear pathway in order to be translated into recognizable behavioral and cognitive patterns, such as personal strengths, to have an effect on self-evaluations of mental health. Simply put, workers need ways to express meaning into actions that benefit their perceptions of mental health. Study 3 shed light on new pathways through which workers may translate the meaning of their into action by cultivating knowledge and understanding of their own personal strengths. This may allow them to apply signature strengths and, in turn, experience vigor, dedication and absorption in their jobs, and to achieve higher levels of mental health.

Overall, the presented studies contribute to the theoretical understanding of the potential value and benefits of strengths knowledge and strengths use in organizations by exploring the application of individual strengths in new cultural Spanish-speaking contexts, offering empirical support for the positive influence of strengths knowledge and strengths use on work-related outcomes (i.e., work engagement, mental health, psychological capital, in-and extra-role performance), understanding the role of strengths use as psychological mechanism that explains the path from meaningful work to well-being (i.e., work engagement, mental health), and the predicting role of meaningful work on strengths use through strengths knowledge. Our main contribution is to support the ability to be aware of and apply signature strengths, as an effective and novel pathway to foster well-being and mental health at work through the cultivation of meaningful work, particularly in times of material uncertainty and change.

### 15.1. Practical implications

This study contributes to the effective assessment of strengths use and strengths knowledge constructs with two validated and reliable instruments. These can also be used to assess and develop effective and high-quality strengths-based interventions within the organizational context in the Spanish-speaking population. Gaining awareness of personal strengths is a necessary precondition toward applying them (Duan et al., 2019). Therefore, it is important to assess both strengths knowledge and strengths use in order to address and apply strengths in work settings. The assessment tools provided in this study could be used as a starting point for employee development in organizations, as an easy-to-implement intervention strategy, not only for identifying strengths, but also defining a plan for developing and using key strengths at work. Previous research sustained that relatively little resources and effort in strengths use interventions can lead to positive outcomes such as employee well-being and productivity (Virga et al., 2022). Together with implementing the SKS and SUS instruments for assessing and developing employees’ strengths, both scales were validated and the theoretical contributions from this study could help organizations and companies to adopt and promote a vision and culture, oriented toward strengths (Bakker and van Woerkom, 2018). Personal strengths assessment could also be considered for recruitment. Previous research has addressed character strengths as predictors for future job performance of potential job candidates (Harzer et al., 2021). Thus, focusing on both identifying and developing personal strengths should be considered an important tool in personal and organizational development for organizations that wish to become healthy and productive, especially in the current era, characterized by material uncertainty and change (Ruch et al., 2020; Salanova, 2020).

Another practical implication of our study is related to the powerful role that meaningful work has in fostering motivation to discover and apply personal strengths. For this reason, we strongly suggest practitioners and HR professionals to focus their attention on stimulating working conditions that can offer employees a greater sense of meaning, for instance by aligning the objectives of their work to their intrinsic values and beliefs, and by fostering top managers to clearly communicate organizational goals, values, and contributions to their employees In this respect, the top managers should play a crucial role in the cultivation of meaningful work among their employees (Van Wingerden and Van der Stoep, 2018). Furthermore, a recent review suggested that, to enable employees to construct their sense of meaningful work, organizations should design effective work environments characterized by well-designed quality jobs, facilitative leaders, cultures, policies and practices, high-quality relationships, and access to decent work. A comprehensive understanding of meaningful work that integrates perspectives across the individual, the organizational, and the societal levels will provide specific propositions about how these levels interact with creating meaningfulness at work (Lysova et al., 2019). Overall, focusing on the development of working conditions that are meaningful leads to a motivation process toward discovering and applying strengths, from which vigor, dedication and absorption in the job arise, and higher levels of mental health are achieved. This assumption is in line with the general notion that the subjective perception of work predicts behavior and attitudes, and thus leads to consequences on perceived well-being and subsequent performance (Van Wingerden and Poell, 2017).

### 15.2. Limitations and future studies

The present study also has some limitations. First, the seven Spanish-speaking countries, considered in the studies, may not be representative of all the countries where Spanish is the primary language. Thus, it might be interesting to replicate the results with a more representative and diversified sample to continue establishing the validity of the scales. Second, strengths knowledge was not used in Study 1, and strengths use was not used in Study 2, thus reliability analysis between these two constructs was not tested. However, Study 3 showed a positive and high correlation among the two variables. Third, data in our studies were cross-sectional, which does not allow to draw firm conclusions about the causal relationship among the variables. There is a need for longitudinal studies to strengthen causal inferences about the influence of meaningful work, strengths use, and strengths knowledge on work-related outcomes.

Future studies could add and explore other variables in our research model. For instance, efforts could be made to analyze the moderating role of leadership styles on the link between meaningful work and strengths, as well as the type of strengths (i.e., optimism, gratitude, etc.) that exert more effects as underlying mechanisms in the path from meaningful work to well-being-related variables. Finally, future studies should strive to identify and include potential “contaminating variables” (Spector et al., 2019) that can also have distinct effects and interact with strengths knowledge and strengths use. This can be done by means of intensive repeated measures designs, such as diary studies including variables, closely related to engagement and mental health, such as positive affect and other well-researched personal resources.

## Data availability statement

The raw data supporting the conclusions of this article will be made available by the authors, without undue reservation.

## Ethics statement

The studies involving human participants were reviewed and approved by Comisión Deontológica de la Universitat Jaume I de Castellón de la Plana. The patients/participants provided their written informed consent to participate in this study.

## Author contributions

JPZ coordinated the research plan process and performed the data collection. JPZ and CCC developed the study design, conducted the analyzes and interpretation of the results, and wrote the manuscript. MS contributed during the development of the study design and revised the manuscript. All the authors listed have made a substantial intellectual contribution to the research and conceived the idea for the study.

## Funding

This study was granted by Generalitat Valenciana (Prometeo [2020/030]).

## Conflict of interest

The authors declare that the research was conducted in the absence of any commercial or financial relationships that could be construed as a potential conflict of interest.

## Publisher’s note

All claims expressed in this article are solely those of the authors and do not necessarily represent those of their affiliated organizations, or those of the publisher, the editors and the reviewers. Any product that may be evaluated in this article, or claim that may be made by its manufacturer, is not guaranteed or endorsed by the publisher.

## Abbreviations

*VIA*-IS: Values in Action-Inventory of Strengths
SKS: Strengths Knowledge Scale
SUS: Strengths Use Scale
JD-R: Job Demands-Resources
PCQ: Psychological Capital Questionnaire
UWES: Utrecht Work Engagement Scale
HERO: HEalthy and Resilient Organizations
GDPR: General Data Protection Regulation
EFA: Exploratory Factor Analysis
CFA: Confirmatory Factor Analysis
*χ* ^2^: chi–square
CFI: Comparative Fit Index
RMSEA: Root Mean Square Error of Approximation
SRMR: Standardized Root Mean Residual
M: Model
GHQ: General Health Questionnaire
KMO: Kaiser-Meyer-Olkin
IFI: Incremental Fit Index
AIC: Akaike Information Criterion
