# Corrigendum: Know and use your personal strengths! A Spanish validation of the strengths knowledge and use scales and their relationship with meaningful work and work-related well-being

**DOI:** 10.3389/fpsyg.2024.1428999

**Published:** 2024-06-18

**Authors:** Josefina Peláez Zuberbühler, Cristián Coo Calcagni, Marisa Salanova

**Affiliations:** WANT Research Team, Universitat Jaume I, Castelló de La Plana, Spain

**Keywords:** personal strengths, meaningful work, work engagement, mental health, well-being, scale validation

In the published article, there was an error in the Funding statement. The funders mentioned in the published article were incorrect. Instead of “Universitat Jaume I [UJI-B2020-08] and the Ministry of Science and Innovation [PID2020-119993RB-I00],” it should be “Generalitat Valenciana (Prometeo [2020/030]).” The corrected Funding statement appears below.

